# Conservative Management of LVAD-Associated Ventricular Pseudoaneurysm

**DOI:** 10.14797/mdcvj.1301

**Published:** 2024-01-18

**Authors:** Marvin Kajy, Connor C. Kerndt, Paul C. Weber, Marzia Leacche, Renzo Y. Loyaga Rendon

**Affiliations:** 1 Corewell Health/Michigan State University, Grand Rapids, Michigan, US

**Keywords:** left ventricular assist device, heart assist device, pseudoaneurysm, advanced heart failure, HeartMate-III left ventricular assist device

## Abstract

Left ventricular assist devices (LVAD) are surgically implanted mechanical support devices utilized with increasing frequency as a bridge to myocardial recovery, destination therapy, and heart transplantation. While the use of such devices in patients with advanced heart failure has shown significant survival benefits and improved quality of life, they bear their own risks and complications.^1^ Bleeding, infection, pump thrombosis, and stroke are just some of the serious complications associated with LVADs.^2^ LVAD-associated pseudoaneurysms are rare, with prior reports of occurrence at the left ventricular apex and at the anastomosis site of the outflow graft to the ascending aorta.^3,4^ Typically, this device-related complication requires surgical repair and, if at all feasible, heart transplantation. However, in cases of difficult anatomy, unfavorable position, and significant comorbidities, surgery may be contraindicated due to high surgical risk. This case portrays a patient suffering from a left ventricular pseudoaneurysm after HeartMate-III implantation that was not amenable to surgical repair due to heightened surgical risk. We document the first pseudoaneurysm associated with the HeartMate-III in available literature and describe a novel management strategy of documented nonoperative course of LVAD-associated pseudoaneurysm, with the patient surviving 56+ months with medical optimization and management.

## Introduction

With technological advances, left ventricular assist devices (LVADs) have proven to be a useful method of mechanical circulatory support as a bridge to transplant or destination therapy in the setting of end-stage heart failure .^2^ Currently, more than 3,200 LVADS are now implanted annually, a number that has only increased over time.^5^ However, with advanced therapies and devices, keeping a vigilant eye for complications becomes increasingly imperative for prompt diagnosis. LVAD therapy commonly implicates risk of bleeding, acquired von Willebrand disease, device thrombosis, systemic thrombosis, device-related infection, worsening valvular disease, ventricular arrhythmia, worsening heart failure, stroke, and hemolysis.^6^ In addition to physiologic complications, the device can also have technical complications including device malpositioning, outflow obstruction, device disconnection, driveline damage, or device failure. Complications related to implantation and device have also been noted, including pseudoaneurysm at the outflow site or ventricular pseudoaneurysm as in this patient.

## Case Report

A gentleman in his thirties with end-stage nonischemic dilated cardiomyopathy received a HeartMate-II left ventricular assist device (Thoratec Corporation) in 2016. His LVAD course was complicated by multiple episodes of suspected and confirmed pump thromboses, requiring a device exchange with a HeartMate-II in 2017. Secondary to a recurrent pump thrombosis in 2018, the patient underwent a pump exchange for a HeartMate-III via left thoracotomy. His postoperative course was complicated by significant surgical site infection requiring multiple wound debridement and left chest wall reconstruction via omental pedicle flap.

**Figure 1:**
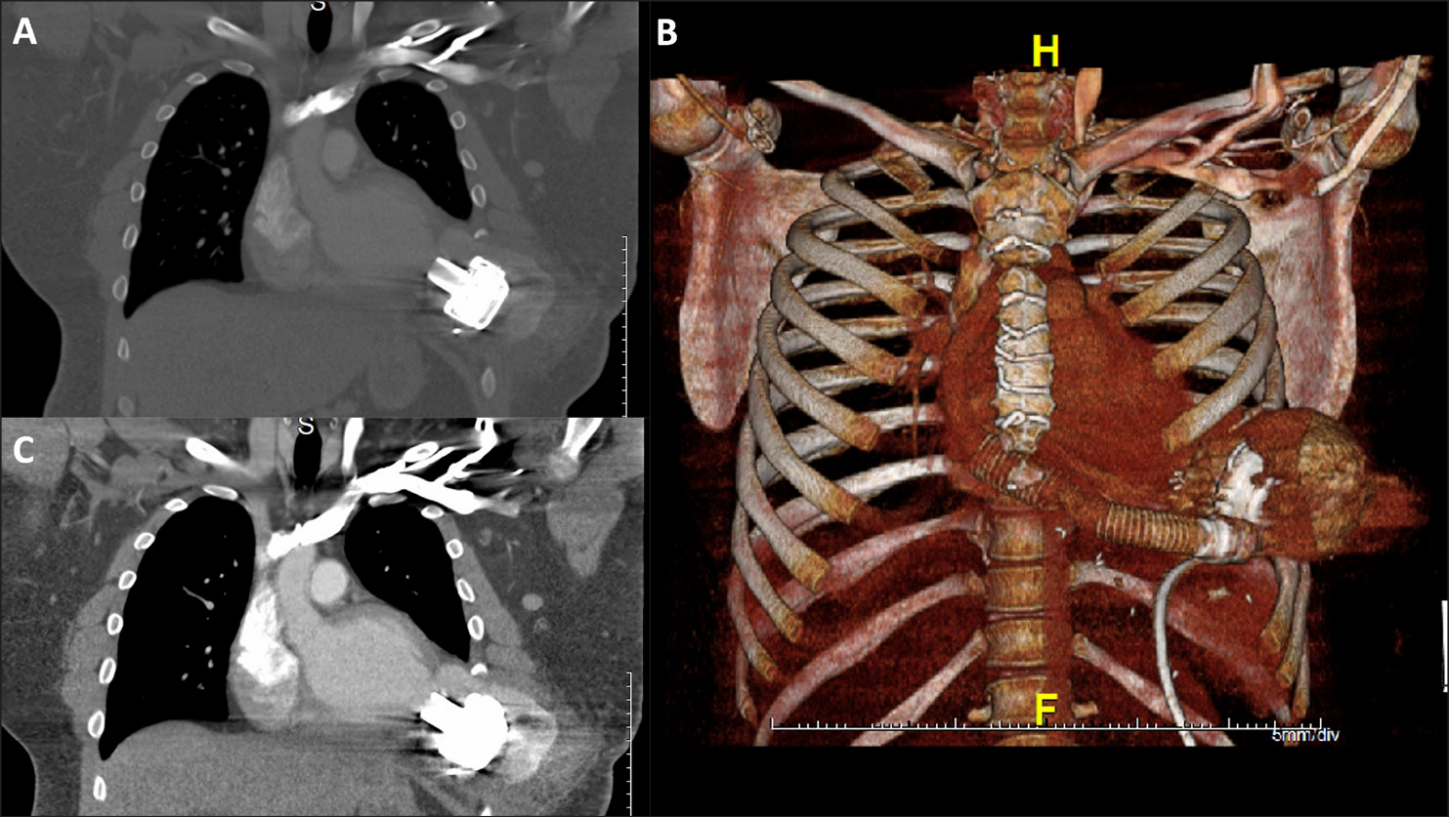
Computed tomography scan demonstrated a large left ventricular pseudoaneurysm measuring 10 × 10 × 8 cm in diameter surrounded by a hematoma. Coronal **(A, C)** and 3-dimensional reconstructed computed tomography **(B)** are shown. H: head; F: front

In 2019, the patient presented to a routine clinic appointment with a chief complaint of a pulsatile chest mass (Video 1). He reported a nontender enlarging ventral hernia over the course of 1 month that subsequently developed into a pulsatile mass. The patient was admitted to the hospital for further evaluation and remained afebrile, with blood cultures showing no evidence of bacteremia. A computed tomography scan of the thorax and abdomen demonstrated a large left ventricular pseudoaneurysm measuring 10 × 10 × 8 cm in diameter surrounded by a hematoma (Figure 1). Transesophageal echocardiogram demonstrated a severely dilated ventricle and a large pseudoaneurysm with a 1.2 cm neck diameter. Color Doppler demonstrated flow in the LVAD and at the pseudoaneurysm neck (Video 1). At the time of diagnosis, the left ventricular diastolic diameter (LVDD) was 7.2 cm, the aortic valve (AV) opened with every beat, the LVAD speed was 5900 rpm, LVAD flow was 5.5 L, power was 5.5 watts, and pulsatility index (PI) was 2.8. Initial medical therapy included losartan 50 mg daily and metoprolol succinate 75 mg daily.

**Video 1:** Transesophageal echocardiogram short axis view of a pulsating left ventricular assist device (LVAD) pseudoaneurysm. Color Doppler demonstrates flow in the LVAD and at the neck of the pseudoaneurysm; see also at https://youtu.be/DSg_b3WFqbg.

Under echocardiographic guidance, the LVAD speed was adjusted to 6300 rpm, flow 6 L, power 5.7 watts, and PI 2.8. Medical therapy was optimized to hydralazine 100 mg 3 times daily, losartan 50 mg daily, metoprolol succinate 50 mg twice daily, amlodipine 5 mg twice daily targeting a mean arterial pressure ~ 65 mm Hg. International normalized ratio goal was not modified (2 - 2.5). After maximal LVAD and medical optimization, echocardiogram showed improvement of LVDD to 6.6 cm, and AV opened with every beat. The patient was not a transplant candidate, and surgical or percutaneous repair were not advised due to extreme surgical risk. Secondary opinions were recommended although not pursued as he elected for pure conservative management. Medical management and surveillance has occurred in the outpatient setting and has demonstrated 92-month survival since diagnosis with New York Heart Association-Functional Classification II/III.

## Discussion

In this case, a male in his thirties with a history of nonischemic cardiomyopathy and LVAD complicated by multiple episodes of pump thrombosis presented with a pulsatile chest-wall mass. Computed tomography imaging demonstrated LVAD-associated pseudoaneurysm with associated intrathoracic hematoma. The patient was treated with medical management due to extreme risk of surgical intervention.

LVAD-associated pseudoaneurysms are rare and have been reported to occur at the LV apex and at the site of outflow graft anastomosis.^7^ Surgical repair poses substantial challenges associated with increased risks due to the patient’s compromised physiology, altered anatomy, and potential mobilization of intracardiac thrombotic material. However, given the rarity of this complication, limited data exists to quantify this risk.^4^ Traditionally, LVAD pseudoaneurysms are repaired through an open approach with repeat sternotomy and selective cardiopulmonary bypass. However, given the inherent risks of repeat sternotomies, Balceniuk et al. pursued a case of a HeartMate-III pseudoaneurysm repair via endovascular repair.^3^

In our patient, due to the proximity and the large dimensions of the pseudoaneurysm, percutaneous closure was thought to be prohibitive. The pseudoaneurysm in this case would have required open repair under deep hypothermia and circulatory arrest. After a multidisciplinary discussion with input from cardiothoracic surgery, advanced heart failure, cardiothoracic critical care, and cardiac anesthesia teams, multiple concerns with surgical repair were identified.

First, in the setting of multiple redo sternotomies and three prior LVAD implants, the mediastinum would have proved hostile and dissection very difficult to perform given significant post-surgical changes. Second, obtaining access to the inflow cannula would have required traversing the anterior abdominal/chest wall hematoma, which likely was providing external pressure to tamponade the pseudoaneurysm. Manipulation to this abdominal hematoma would risk exsanguination. Given the patient’s history of multiple prior sternotomies and multiple debridement of the left thoracotomy site, including reconstruction with an omental flap, his surgical risk was considered to be prohibitive.

Heart transplantation potentially could have been the ideal treatment, but unfortunately the patient did not meet criteria for transplantation at our institution due to his body mass index and noncompliance. The patient declined surgical evaluation at another center and decided to proceed with medical management. Unfortunately, due to the rarity of LVAD-associated pseudoaneurysms, optimal therapy and optimization of LVAD settings have not been described or studied to date. The approach pursued for this patient’s medical management was to unload the ventricle and reduce afterload. Antihypertensive medications were optimized to target a MAP goal of 65 mm Hg to decrease left ventricular wall stress. Additionally, our team chose to increase LVAD pump speed to decompress and unload the left ventricle and theoretically reduce blood flow in the pseudoaneurysm. Over time, the left ventricular pseudoaneurysm has increased in size, but the patient has remained stable and followed in our clinic. This case illustrates ongoing challenges in the field of end stage heart failure and mechanical circulatory support, and how under specific circumstances conservative management, although counterintuitive, may produce the best long-term survival and quality of life for a patient.

Current data of LVAD-associated pseudoaneurysm management is limited to case reports. Studies are needed to further characterize the long-term survival rates and complications associated with traditional surgical intervention compared with medical management.

## Conclusion

This case portrays the first documented pseudoaneurysm associated with the HeartMate-III LVAD. Our case demonstrates the novel management of LVAD-associated pseudoaneurysm with conservative measures given the substantial risk of any surgical intervention.
